# Supplementary material for: A Likelihood Approach for Real-Time Calibration of Stochastic Compartmental Epidemic Models
Source: PLoS Comput Biol. 2017 Jan 17;13(1):e1005257. doi: 10.1371/journal.pcbi.1005257 (PMC5240920; doi:10.1371/journal.pcbi.1005257)
Supplement: S1 File — (TAR.GZ) [file pcbi.1005257.s014.tar.gz › HSPH_Online-SI-Revision/IPoi09/n1-mild/n1-mild_BENCH2_table4.pdf]

1	newly 3 weeks spec	6.	31.7455	{12., 54.}	429.091	0.666962	newly 3 weeks cumul	40.	111.109	{49., 174.}	179.227	0.409703	newly 5 weeks spec	2.	22.9455	{8., 45.}	1047.27	0.967846	newly 5 weeks cumul	43.	160.927	{76., 255.}	274.249	0.526286	newly 51 weeks cumul	116.	252.073	{101., 442.}	124.201	0.308677
2	newly 3 weeks spec	3.	6.5303	{1., 19.}	159.091	0.448104	newly 3 weeks cumul	16.	24.5303	{5., 64.}	96.875	0.319376	newly 5 weeks spec	4.	3.36364	{0., 8.}	69.697	0.528847	newly 5 weeks cumul	23.	32.0455	{8., 90.}	86.8906	0.30888	newly 47 weeks cumul	28.	43.8788	{8., 129.}	98.3766	0.319341
3	newly 3 weeks spec	13.	2.71429	{0., 11.}	84.0659	1.35203	newly 3 weeks cumul	35.	15.0357	{0., 41.}	73.5714	0.976686	newly 5 weeks spec	6.	1.53571	{0., 6.}	89.881	1.39915	newly 5 weeks cumul	61.	19.1429	{0., 48.}	78.9227	1.14882	newly 56 weeks cumul	77.	20.9643	{0., 54.}	81.0297	1.23068
4	newly 3 weeks spec	9.	9.175	{1., 18.}	69.7222	0.41815	newly 3 weeks cumul	30.	33.525	{7., 63.}	65.25	0.293507	newly 5 weeks spec	8.	5.45	{1., 12.}	66.875	0.545827	newly 5 weeks cumul	45.	46.45	{9., 97.}	64.	0.312375	newly 50 weeks cumul	300.	71.4	{9., 141.}	76.2	0.79037
5	newly 3 weeks spec	10.	22.5714	{8., 42.}	134.603	0.301173	newly 3 weeks cumul	42.	86.0317	{37., 144.}	109.524	0.268338	newly 5 weeks spec	5.	12.9524	{5., 26.}	169.206	0.345965	newly 5 weeks cumul	52.	115.095	{49., 182.}	125.	0.289848	newly 52 weeks cumul	70.	151.952	{58., 229.}	123.061	0.283788
6	newly 3 weeks spec	20.	58.0357	{32., 83.}	190.357	0.439004	newly 3 weeks cumul	66.	189.429	{120., 244.}	187.013	0.447117	newly 5 weeks spec	12.	42.1607	{21., 67.}	253.423	0.514953	newly 5 weeks cumul	97.	280.036	{168., 363.}	188.697	0.445559	newly 56 weeks cumul	201.	433.518	{229., 612.}	117.617	0.317313
7	newly 3 weeks spec	20.	24.8475	{6., 58.}	71.5254	0.287743	newly 3 weeks cumul	60.	95.5085	{32., 202.}	80.0847	0.238985	newly 5 weeks spec	5.	14.2712	{3., 29.}	201.695	0.400498	newly 5 weeks cumul	86.	128.356	{42., 273.}	76.7245	0.245105	newly 55 weeks cumul	126.	174.356	{47., 358.}	78.0334	0.263093
8	newly 3 weeks spec	10.	24.5714	{13., 43.}	149.388	0.369729	newly 3 weeks cumul	44.	80.6939	{44., 123.}	89.7032	0.267537	newly 5 weeks spec	12.	20.6122	{8., 32.}	82.6531	0.250803	newly 5 weeks cumul	74.	123.918	{68., 180.}	73.8555	0.228751	newly 47 weeks cumul	324.	302.918	{139., 557.}	40.4825	0.198001
9	newly 3 weeks spec	13.	25.5091	{11., 49.}	102.657	0.276261	newly 3 weeks cumul	57.	87.0727	{41., 138.}	63.9872	0.204292	newly 5 weeks spec	5.	19.0909	{7., 37.}	281.818	0.503679	newly 5 weeks cumul	72.	128.255	{62., 234.}	85.7071	0.24416	newly 46 weeks cumul	74.	224.491	{108., 415.}	204.3	0.424119
10	newly 3 weeks spec	5.	13.4328	{5., 28.}	177.015	0.376952	newly 3 weeks cumul	23.	49.403	{22., 99.}	121.025	0.288927	newly 5 weeks spec	1.	8.16418	{2., 20.}	725.373	0.804918	newly 5 weeks cumul	26.	68.1343	{31., 128.}	165.844	0.357673	newly 47 weeks cumul	26.	101.821	{40., 197.}	292.652	0.50521
11	newly 3 weeks spec	6.	28.8478	{17., 40.}	380.797	0.655228	newly 3 weeks cumul	21.	89.7609	{50., 124.}	327.433	0.607878	newly 5 weeks spec	11.	24.3913	{12., 38.}	124.901	0.315305	newly 5 weeks cumul	43.	139.978	{86., 190.}	225.531	0.488715	newly 46 weeks cumul	315.	343.543	{165., 573.}	37.8951	0.159672
12	newly 3 weeks spec	9.	28.1591	{11., 44.}	217.929	0.471665	newly 3 weeks cumul	42.	86.5682	{44., 122.}	111.634	0.312075	newly 5 weeks spec	9.	22.8409	{11., 36.}	157.828	0.376314	newly 5 weeks cumul	57.	136.136	{71., 196.}	142.743	0.36492	newly 47 weeks cumul	206.	327.205	{186., 526.}	68.7004	0.219362
13	newly 3 weeks spec	6.	7.31579	{1., 20.}	88.5965	0.436275	newly 3 weeks cumul	20.	31.3158	{8., 60.}	92.7193	0.288223	newly 5 weeks spec	0.1	4.94737	{0., 13.}	4875.44	1.36813	newly 5 weeks cumul	21.	42.3509	{11., 74.}	126.566	0.311956	newly 48 weeks cumul	26.	56.4737	{11., 102.}	145.007	0.339195
14	newly 3 weeks spec	4.	18.6842	{5., 38.}	375.	0.598382	newly 3 weeks cumul	23.	76.0877	{29., 149.}	235.393	0.461573	newly 5 weeks spec	3.	10.807	{1., 23.}	277.778	0.535358	newly 5 weeks cumul	29.	102.439	{41., 195.}	257.108	0.482825	newly 52 weeks cumul	29.	132.439	{47., 268.}	359.347	0.574712
15	newly 3 weeks spec	19.	22.1702	{5., 45.}	59.2385	0.262441	newly 3 weeks cumul	57.	87.0638	{35., 159.}	71.8552	0.230542	newly 5 weeks spec	13.	16.1489	{3., 36.}	78.2324	0.320536	newly 5 weeks cumul	84.	121.979	{40., 219.}	71.4032	0.240314	newly 52 weeks cumul	175.	169.	{50., 309.}	52.5471	0.264931
16	newly 3 weeks spec	24.	13.46	{2., 28.}	55.5833	0.526039	newly 3 weeks cumul	51.	50.6	{11., 102.}	60.0784	0.329123	newly 5 weeks spec	13.	7.92	{0., 18.}	61.5385	0.618129	newly 5 weeks cumul	79.	69.1	{13., 146.}	58.	0.363122	newly 50 weeks cumul	230.	93.36	{13., 214.}	63.0609	0.598069
17	newly 3 weeks spec	19.	24.7273	{9., 49.}	61.3397	0.26096	newly 3 weeks cumul	42.	84.9273	{30., 156.}	116.061	0.347492	newly 5 weeks spec	16.	17.6545	{4., 36.}	70.5682	0.348056	newly 5 weeks cumul	69.	124.8	{41., 240.}	99.2622	0.321837	newly 53 weeks cumul	203.	201.364	{55., 390.}	55.3515	0.314026
18	newly 3 weeks spec	17.	27.2963	{11., 38.}	73.6383	0.243667	newly 3 weeks cumul	55.	91.1852	{48., 130.}	73.8047	0.234708	newly 5 weeks spec	17.	21.9815	{9., 36.}	52.8322	0.20281	newly 5 weeks cumul	88.	140.352	{76., 184.}	68.4975	0.222223	newly 46 weeks cumul	176.	286.852	{117., 453.}	77.6726	0.240569
19	newly 3 weeks spec	22.	17.1774	{5., 32.}	55.7918	0.340693	newly 3 weeks cumul	64.	70.4516	{25., 124.}	51.2601	0.216653	newly 5 weeks spec	12.	10.9516	{2., 17.}	64.3817	0.370438	newly 5 weeks cumul	88.	95.7903	{30., 163.}	54.0506	0.230931	newly 53 weeks cumul	114.	125.79	{31., 208.}	61.0215	0.256907
20	newly 3 weeks spec	10.	30.6379	{14., 53.}	207.069	0.437201	newly 3 weeks cumul	38.	106.345	{57., 161.}	179.855	0.414697	newly 5 weeks spec	11.	20.7931	{6., 42.}	110.031	0.294299	newly 5 weeks cumul	53.	152.897	{76., 243.}	188.549	0.419988	newly 50 weeks cumul	69.	246.121	{110., 420.}	256.997	0.489504
21	newly 3 weeks spec	11.	6.17391	{1., 13.}	58.8933	0.488576	newly 3 weeks cumul	47.	28.1087	{7., 63.}	56.383	0.415919	newly 5 weeks spec	5.	3.78261	{0., 9.}	66.9565	0.580773	newly 5 weeks cumul	63.	37.7174	{10., 82.}	55.176	0.414372	newly 50 weeks cumul	68.	47.3478	{10., 95.}	54.9233	0.385026
22	newly 3 weeks spec	16.	23.1923	{7., 41.}	70.6731	0.275436	newly 3 weeks cumul	38.	81.5385	{36., 129.}	122.773	0.327812	newly 5 weeks spec	16.	16.8077	{4., 30.}	53.6058	0.298102	newly 5 weeks cumul	74.	118.635	{48., 194.}	76.5333	0.252088	newly 52 weeks cumul	332.	203.135	{79., 374.}	48.1406	0.323093
23	newly 3 weeks spec	14.	8.18605	{1., 17.}	65.7807	0.520888	newly 3 weeks cumul	59.	30.7209	{9., 65.}	60.5439	0.452945	newly 5 weeks spec	8.	5.62791	{1., 13.}	75.5814	0.587691	newly 5 weeks cumul	90.	43.093	{13., 91.}	63.7468	0.498798	newly 47 weeks cumul	150.	64.0233	{16., 149.}	67.9845	0.580799
24	newly 3 weeks spec	16.	32.7705	{16., 52.}	111.578	0.301253	newly 3 weeks cumul	68.	106.754	{58., 158.}	64.5612	0.205101	newly 5 weeks spec	13.	25.8033	{11., 49.}	109.584	0.280973	newly 5 weeks cumul	98.	162.23	{94., 247.}	72.5661	0.219056	newly 49 weeks cumul	197.	307.311	{157., 558.}	70.8746	0.215192
25	newly 3 weeks spec	13.	16.6939	{3., 34.}	74.8823	0.314377	newly 3 weeks cumul	29.	59.5714	{21., 110.}	117.382	0.2993	newly 5 weeks spec	8.	12.7347	{3., 32.}	106.633	0.34913	newly 5 weeks cumul	44.	87.3878	{29., 176.}	115.306	0.299367	newly 46 weeks cumul	191.	163.776	{41., 379.}	60.6475	0.353532
26	newly 3 weeks spec	5.	23.2712	{8., 43.}	368.814	0.59148	newly 3 weeks cumul	24.	82.4576	{35., 141.}	243.573	0.479726	newly 5 weeks spec	1.	17.2712	{5., 36.}	1627.12	1.10786	newly 5 weeks cumul	34.	119.644	{50., 207.}	251.894	0.486516	newly 49 weeks cumul	44.	202.051	{74., 435.}	359.206	0.565785
27	newly 3 weeks spec	19.	24.5273	{12., 39.}	45.3589	0.169522	newly 3 weeks cumul	39.	86.5091	{43., 125.}	122.657	0.324397	newly 5 weeks spec	10.	19.6364	{9., 36.}	105.455	0.29573	newly 5 weeks cumul	66.	130.236	{58., 178.}	101.791	0.289109	newly 46 weeks cumul	107.	263.564	{109., 377.}	150.229	0.362257
28	newly 3 weeks spec	12.	33.88	{17., 52.}	190.667	0.47909	newly 3 weeks cumul	35.	107.26	{54., 148.}	214.914	0.502194	newly 5 weeks spec	8.	30.46	{13., 45.}	286.75	0.562927	newly 5 weeks cumul	50.	171.22	{107., 239.}	250.12	0.544979	newly 52 weeks cumul	232.	440.44	{252., 634.}	91.7414	0.26166
29	newly 3 weeks spec	13.	22.8571	{9., 49.}	91.4835	0.244271	newly 3 weeks cumul	47.	81.6786	{33., 149.}	84.7264	0.237899	newly 5 weeks spec	5.	14.9286	{4., 27.}	204.286	0.406761	newly 5 weeks cumul	61.	115.161	{53., 209.}	97.3361	0.256935	newly 49 weeks cumul	61.	180.375	{78., 351.}	198.156	0.401162
30	newly 3 weeks spec	22.	15.3519	{5., 28.}	46.0438	0.335292	newly 3 weeks cumul	51.	65.0741	{24., 126.}	60.5664	0.231748	newly 5 weeks spec	11.	8.07407	{3., 16.}	48.1481	0.328217	newly 5 weeks cumul	78.	84.2037	{31., 156.}	50.3561	0.22913	newly 53 weeks cumul	111.	106.815	{39., 199.}	47.3807	0.245742
31	newly 3 weeks spec	4.	25.9342	{12., 43.}	548.355	0.749671	newly 3 weeks cumul	23.	99.75	{49., 164.}	333.696	0.589309	newly 5 weeks spec	0.1	17.2368	{8., 30.}	17136.8	2.15975	newly 5 weeks cumul	29.	137.276	{66., 223.}	373.367	0.622133	newly 51 weeks cumul	29.	197.658	{90., 325.}	581.579	0.768685
32	newly 3 weeks spec	21.	24.2143	{12., 39.}	40.7029	0.205195	newly 3 weeks cumul	44.	77.5714	{39., 125.}	86.1472	0.306262	newly 5 weeks spec	15.	18.2619	{5., 31.}	56.9841	0.274077	newly 5 weeks cumul	72.	116.714	{54., 186.}	75.2646	0.292519	newly 49 weeks cumul	281.	262.81	{100., 495.}	46.4328	0.303673
33	newly 3 weeks spec	13.	34.7	{17., 55.}	168.242	0.392481	newly 3 weeks cumul	55.	118.014	{63., 174.}	115.662	0.309009	newly 5 weeks spec	7.	23.8571	{9., 40.}	242.041	0.482039	newly 5 weeks cumul	72.	170.614	{82., 258.}	137.242	0.343846	newly 50 weeks cumul	85.	280.914	{115., 463.}	230.487	0.471116
34	newly 3 weeks spec	12.	7.57143	{0., 16.}	68.1973	0.679214	newly 3 weeks cumul	36.	27.3469	{1., 58.}	61.5646	0.528652	newly 5 weeks spec	15.	4.5102	{0., 11.}	79.7279	1.1061	newly 5 weeks cumul	60.	38.0408	{1., 85.}	64.2857	0.611077	newly 49 weeks cumul	138.	50.5714	{1., 107.}	72.5525	0.842981
35	newly 3 weeks spec	15.	40.6866	{19., 66.}	174.03	0.404045	newly 3 weeks cumul	76.	137.91	{78., 201.}	84.4462	0.250218	newly 5 weeks spec	15.	28.	{12., 50.}	97.6119	0.271504	newly 5 weeks cumul	115.	199.701	{105., 302.}	78.4815	0.23608	newly 51 weeks cumul	153.	316.179	{152., 513.}	109.521	0.291095
36	newly 3 weeks spec	2.	19.7031	{7., 36.}	885.156	0.908061	newly 3 weeks cumul	28.	78.0156	{35., 123.}	179.632	0.398956	newly 5 weeks spec	0.1	10.2188	{2., 18.}	10118.7	1.8904	newly 5 weeks cumul	28.	103.891	{47., 165.}	271.261	0.515452	newly 53 weeks cumul	28.	130.938	{59., 208.}	367.634	0.606885
37	newly 3 weeks spec	11.	17.1552	{6., 31.}	80.721	0.244596	newly 3 weeks cumul	35.	58.9138	{21., 104.}	85.9606	0.249887	newly 5 weeks spec	8.	11.8448	{2., 24.}	89.4397	0.333711	newly 5 weeks cumul	53.	84.6724	{28., 168.}	82.661	0.251118	newly 49 weeks cumul	95.	142.293	{41., 282.}	84.7005	0.27325
38	newly 3 weeks spec	9.	21.2714	{8., 42.}	146.19	0.345237	newly 3 weeks cumul	41.	83.0429	{32., 142.}	112.16	0.283172	newly 5 weeks spec	5.	12.5143	{3., 22.}	169.714	0.384854	newly 5 weeks cumul	58.	112.014	{43., 188.}	103.67	0.264137	newly 53 weeks cumul	65.	149.9	{50., 251.}	138.747	0.31289
39	newly 3 weeks spec	13.	9.43396	{1., 20.}	63.135	0.500553	newly 3 weeks cumul	39.	37.0755	{8., 70.}	58.7325	0.308376	newly 5 weeks spec	4.	5.43396	{0., 11.}	99.0566	0.475885	newly 5 weeks cumul	49.	49.3774	{12., 95.}	63.2268	0.315908	newly 51 weeks cumul	78.	63.8302	{13., 127.}	60.7402	0.36495
40	newly 3 weeks spec	11.	20.0167	{6., 41.}	101.667	0.261243	newly 3 weeks cumul	36.	81.0833	{35., 158.}	129.954	0.307424	newly 5 weeks spec	1.	11.4	{3., 24.}	1043.33	0.904113	newly 5 weeks cumul	43.	107.817	{44., 214.}	154.225	0.338709	newly 54 weeks cumul	43.	139.517	{53., 256.}	226.705	0.423385
41	newly 3 weeks spec	9.	16.0345	{3., 36.}	113.41	0.34344	newly 3 weeks cumul	33.	72.5	{21., 142.}	135.789	0.327875	newly 5 weeks spec	5.	8.2931	{0., 20.}	121.034	0.508173	newly 5 weeks cumul	42.	91.8793	{25., 186.}	138.136	0.341699	newly 55 weeks cumul	48.	120.414	{25., 231.}	172.414	0.374229
42	newly 3 weeks spec	0.1	23.9077	{7., 52.}	23807.7	2.27341	newly 3 weeks cumul	0.1	93.8462	{36., 188.}	93746.2	2.90357	newly 5 weeks spec	0.1	12.9692	{3., 28.}	12869.2	1.95554	newly 5 weeks cumul	0.1	124.631	{45., 247.}	124531.	3.01737	newly 54 weeks cumul	0.1	154.631	{54., 322.}	154531.	3.10158
43	newly 3 weeks spec	2.	9.84507	{3., 19.}	395.07	0.558988	newly 3 weeks cumul	19.	49.507	{18., 82.}	166.938	0.341349	newly 5 weeks spec	2.	3.77465	{0., 8.}	139.437	0.498495	newly 5 weeks cumul	24.	59.338	{21., 96.}	156.279	0.330283	newly 56 weeks cumul	27.	65.9437	{21., 118.}	154.564	0.325277
44	newly 3 weeks spec	2.	23.2188	{12., 41.}	1060.94	1.00946	newly 3 weeks cumul	12.	82.5313	{35., 130.}	587.76	0.794438	newly 5 weeks spec	0.1	16.9063	{6., 34.}	16806.2	2.11868	newly 5 weeks cumul	14.	118.016	{48., 191.}	742.969	0.876427	newly 48 weeks cumul	17.	193.969	{75., 320.}	1040.99	0.985998
45	newly 3 weeks spec	13.	14.6333	{6., 27.}	51.7949	0.213753	newly 3 weeks cumul	46.	55.35	{21., 99.}	49.3841	0.197081	newly 5 weeks spec	7.	9.76667	{3., 19.}	67.1429	0.238857	newly 5 weeks cumul	63.	76.9	{30., 134.}	48.7831	0.188916	newly 46 weeks cumul	86.	131.15	{47., 214.}	71.1047	0.206479
46	newly 3 weeks spec	11.	18.4318	{3., 33.}	96.9008	0.345772	newly 3 weeks cumul	54.	67.4318	{19., 118.}	55.4293	0.222756	newly 5 weeks spec	9.	11.5455	{1., 25.}	82.3232	0.464992	newly 5 weeks cumul	77.	94.2727	{22., 168.}	58.5006	0.247341	newly 53 weeks cumul	299.	142.068	{24., 280.}	56.0429	0.469786
47	newly 3 weeks spec	14.	29.6111	{14., 45.}	116.27	0.306128	newly 3 weeks cumul	32.	88.9167	{43., 141.}	180.642	0.4171	newly 5 weeks spec	10.	25.5	{11., 43.}	157.222	0.364551	newly 5 weeks cumul	50.	143.056	{69., 215.}	187.	0.424514	newly 51 weeks cumul	260.	349.139	{187., 532.}	48.515	0.169103
48	newly 3 weeks spec	9.	24.7037	{11., 41.}	178.601	0.401021	newly 3 weeks cumul	23.	84.7222	{40., 138.}	268.357	0.518431	newly 5 weeks spec	3.	18.8519	{6., 38.}	528.395	0.704193	newly 5 weeks cumul	34.	125.352	{62., 234.}	268.682	0.513101	newly 46 weeks cumul	83.	220.87	{100., 431.}	168.251	0.367754
49	newly 3 weeks spec	8.	22.1628	{7., 40.}	182.267	0.391198	newly 3 weeks cumul	37.	73.186	{30., 130.}	106.6	0.282262	newly 5 weeks spec	3.	19.	{3., 33.}	539.535	0.705095	newly 5 weeks cumul	43.	113.581	{38., 197.}	169.767	0.383624	newly 50 weeks cumul	190.	246.651	{61., 502.}	70.1346	0.280566
50	newly 3 weeks spec	19.	23.4844	{10., 42.}	53.2072	0.198378	newly 3 weeks cumul	60.	82.2656	{37., 147.}	56.3281	0.196823	newly 5 weeks spec	13.	16.125	{5., 27.}	60.5769	0.234496	newly 5 weeks cumul	91.	117.328	{54., 187.}	52.0776	0.191786	newly 47 weeks cumul	148.	195.672	{79., 307.}	57.3374	0.196475
